# Clinical Outcome of Induction Treatment in the Era of Novel Agents and the Impact of the Number of High‐Risk Cytogenetic Abnormalities (HRA) on Prognosis of Patients With Newly Diagnosed Multiple Myeloma (NDMM): Insights From a Multicenter Study

**DOI:** 10.1002/cam4.70270

**Published:** 2024-10-18

**Authors:** Dong Liang, Xiaojin Li, Shenrui Bai, Qiaoli Wang, Min Zeng, Demei Feng, Bo Lu, Xiaoqing Li, Zhiqiang Sun, Jianyun Li, Huanhuan Zhou, Jialu Zhang, Xiaoqin Chen, Zhongjun Xia, Yang Liang, Hua Wang

**Affiliations:** ^1^ State Key Laboratory of Oncology in South China, Guangdong Provincial Clinical Research Center for Cancer Sun Yat‐Sen University Cancer Center Guangzhou China; ^2^ Guangdong Provincial Key Laboratory of Digestive Cancer Research, Digestive Diseases Center, Scientific Research Center The Seventh Affiliated Hospital of Sun Yat‐Sen University Shenzhen Guangdong China; ^3^ Hematology Department Shenzhen Second People's Hospital Shenzhen Guangdong China; ^4^ Hematology Department, Shenzhen Hospital Southern Medical University Shenzhen China; ^5^ Hematology Department People's Hospital of Shenzhen Bao an District Shenzhen China; ^6^ Hematology Department Huazhong University of Science and Technology, Union Shenzhen Hospital Shenzhen China; ^7^ Hematology Department The Second Affiliated Hospital of Harbin Medical University Harbin Heilongjiang China

**Keywords:** 1q21 gain/amplification, CD38, high risk cytogenetic abnormalities, newly diagnosed multiple myeloma

## Abstract

**Background:**

In the era of novel agents, the clinical outcomes of induction treatment and the impact of the number of high‐risk cytogenetic abnormalities (HRA) in newly diagnosed multiple myeloma (NDMM) need to be explored.

**Objective:**

Through this study, we aim to analyze the effectiveness of different induction treatments and explore the survival outcomes of patients with varying numbers of HRA.

**Methods:**

A total of 734 patients from seven medical centers were included in our study.

**Results:**

Patients in the CD38 monoclonal antibody or IMiDs plus proteasome inhibitors (PI) groups had significantly superior overall survival (OS) and progression‐free survival (PFS) compared to those receiving IMiDs or PI alone. Additionally, the CD38 monoclonal antibody conferred a PFS advantage over IMiDs plus PI. Patients with ≥ 2 high‐risk cytogenetic abnormalities (HRA) exhibited an extremely poor prognosis and should be considered ultra‐high‐risk individuals in multiple myeloma (MM). The CD38 monoclonal antibody, transplantation, and achieving minimal residual disease (MRD) negativity only partly mitigated the poor prognosis in patients with HRA. Furthermore, patients with 1q21 gain/amplification (1q21+) only had a significantly worse prognosis compared to patients without HRA, and those with 1q21+ plus del17p or t(4;14) exhibited an inferior prognosis compared to those with 1q21+ alone.

**Conclusion:**

Our results suggested that double‐hit multiple myeloma was associated with extremely poor survival outcomes, and more effective treatments needed to be explored for this particular subtype of MM.

## 1 Introduction

As the second most common hematological malignancy, multiple myeloma (MM) has continually been presented as an incurable disease with unfavorable clinical outcomes for patients. In the era of conventional treatment, the 5‐year OS rate for MM patients was disheartening. However, the landscape shifted significantly with the introduction of novel agents, notably proteasome inhibitors (PI) and immunomodulatory agents (IMiDs), which effectively prolonged the time from disease diagnosis to progression and relapse among patients.

A pivotal randomized Phase III clinical trial showcased the superiority of combining IMiDs with PI, as patients receiving bortezomib, lenalidomide, and dexamethasone (VRD) demonstrated markedly improved OS and PFS compared to those receiving lenalidomide and dexamethasone (Rd) alone [1].

Further advancement came with the FDA approval of CD38 monoclonal antibody‐daratumumab in 2019, marking a significant milestone in treatment strategies. Daratumumab‐based regimens emerged as game changers, offering substantially enhanced PFS and deeper treatment responses compared to other therapies. Notably, a randomized Phase III clinical trial illustrated the remarkable efficacy of daratumumab, bortezomib, lenalidomide, and dexamethasone (DVRD) in improving PFS, treatment response, and minimal residual disease (MRD) negativity rates when compared to VRD, solidifying daratumumab's pivotal role in preferred treatment modalities for NDMM [2].

These breakthroughs have propelled significant strides in MM management. It is now intriguing to explore the real‐world outcomes among patients receiving PI or IMiDs based induction treatment, PI plus IMiDs based maintenance, and CD38 monoclonal antibody‐based regimens.

High‐risk cytogenetic abnormalities (HRA) have been identified as risk factors that lead to reductions in overall survival (OS) and progression‐free survival (PFS) for patients with MM [3, 4]. These HRAs include deletions of 17p, 1q21 gain/amplification, t(4;14), and t(14;16). According to the Stratification for Myeloma and Risk‐Adapted Therapy 3.0 (mSMART 3.0) guidelines from the Mayo Clinic, a new definition, double‐hit MM, was proposed [5]. Double‐hit MM refers to the presence of two HRAs, while triple‐hit MM refers to the presence of at least three HRAs. Patients with double‐hit MM are considered ultra‐high‐risk individuals, which confers an extremely poor prognosis [6, 7]. Based on these definitions, it is crucial to explore the clinical outcomes of double‐hit and triple‐hit MM. Additionally, risk‐adapted therapy according to HRAs warrants discussion in clinical practice. Therefore, we conducted a multicenter retrospective study to investigate the real‐world clinical outcomes of frontline therapy and to explore HRAs in newly diagnosed MM (NDMM).

## 2 Methods

Clinical data from Sun Yat‐Sen University Cancer Center were retrospectively analyzed for NDMM patients between 2016 and 2023. May 1, 2024 served as the final follow‐up date. Anonymized data analysis approval was granted by the Institutional Ethical Review Board, which also waived informed consent requirements. Stringent procedures ensured data accuracy and completeness.

Inclusion criteria targeted individuals newly diagnosed with MM from Sun Yat‐Sen University Cancer Center and other hospitals. Patients lost to follow‐up were excluded, resulting in a final analysis of 734 patients.

For transplant‐eligible patients, the standard induction treatment consisted of four cycles followed by transplantation. After transplantation, it was recommended that these patients receive an additional two cycles of induction treatment. Non‐transplant recipients underwent a standard induction treatment of nine cycles. Even if non‐transplant recipients achieved stringent complete response (sCR) after initial induction treatment, they still required at least six cycles of induction. Transplantation for eligible patients could be performed after completion of four cycles of induction treatment. At our center, maintenance treatment was continued until disease progression, patient preference to discontinue maintenance, or the occurrence of intolerable adverse events. Salvage therapy for transplant recipients involved either advising them to participate in clinical trials or adding novel agents such as carfilzomib or daratumumab to their treatment regimen and salvage treatment for non‐transplant recipients followed a similar approach.

High‐risk patients were defined as those carrying at least one cytogenetic abnormality classified as high risk. Standard risk patients were defined as those without HRAs. MRD− was defined as patients who achieved MRD negativity during the treatment course, while MRD+ was defined as patients who had consistent MRD positivity during treatment.

Response to induction treatment was assessed using International Myeloma Working Group (IMWG) criteria [8]. MRD evaluation, conducted throughout treatment via flow cytometry, established the lowest detectable MRD value at 10^‐5~10^‐4 [9]. A *p* value < 0.05 was considered as statistically significant.

### 2.1 Statistical Analysis

Baseline characteristics were compared between treatment groups using Fisher's exact test and the chi‐squared test for categorical variables and the Wilcoxon rank‐sum test or Student's *t*‐test for continuous variables. OS was defined as the time between induction initiation and eventual death from any cause. PFS was the time from induction initiation to progression or death from any cause. Kaplan–Meier curves were used to visualize OS and PFS and log‐rank tests assessed differences among different induction treatment groups. A multivariable Cox proportional hazards model was built to analyze the impact of induction therapy on OS and PFS, accounting for other relevant variables. All analyses were performed using CRAN R Version 4.3.2 (The R Foundation for Statistical Computing, Vienna, Austria).

## 3 Results

In this study, a total of 734 patients were included for final analysis. Of these, 337 patients received IMiDs plus PI for induction treatment, 134 patients received daratumumab‐based induction, and 263 patients received either IMiDs or PI. Among the 529 patients with available FISH results, 327 patients did not have HRA, 156 patients had only one HRA, 37 patients had two HRA, and 9 patients had ≥ 3 HRA. Due to the small number of patients with triple‐hit MM, we combined patients with two HRA and those with ≥ 3 HRA into a single group with ≥ 2 HRA (Table 1).

**TABLE 1:** Baseline characteristics for patients in different induction treatment group.

	IMiDs + PI	CD38	IMiDs or PI	*p*
n	365	134	321	
Sex (%)				0.056
Female	155 (46.0)	49 (36.6)	99 (37.6)	
Male	182 (54.0)	85 (63.4)	164 (62.4)	
Age (%)				0.056
≥ 65	114 (33.8)	51 (38.1)	71 (27.0)	
< 65	223 (66.2)	83 (61.9)	192 (73.0)	
ECOG performance status (%)				0.018
0–1	234 (69.4)	75 (71.4)	156 (59.5)	
≥ 2	103 (30.6)	30 (28.6)	106 (40.5)	
Unknown	0	29	1	
M protein (median [IQR])	15.00 [1.00, 33.00]	17.32 [2.50, 41.50]	14.50 [2.00, 41.00]	0.642
Number of high‐risk cytogenetics (%)				0.872
0–1	238 (90.5)	103 (91.1)	142 (92.8)	
≥ 2	25 (9.5)	10 (8.9)	11 (7.2)	
Unknown	74	21	110	
Transplantation (%)				0.005
Yes	75 (22.3)	37 (27.6)	38 (14.4)	
No	262 (77.7)	97 (72.4)	225 (85.6)	
Risk stratification (%)				0.569
High risk	97 (36.9)	48 (42.5)	57 (37.3)	
Standard risk	166 (63.1)	65 (57.5)	96 (62.7)	
Unknown	74	21	110	
17p‐ (%)	19 (7.2)	5 (4.6)	11 (7.2)	0.62
t(4;14) (%)	20 (7.6)	10 (9.1)	13 (8.5)	0.879
t(14;16) (%)	2 (0.8)	3 (2.7)	3 (2.0)	0.32
1q21+ (%)	86 (32.7)	44 (38.9)	43 (28.1)	0.177
ISS stage (%)				0.268
1	114 (34.5)	41 (31.8)	87 (34.4)	
2	96 (29.1)	32 (24.8)	56 (22.1)	
3	120 (36.4)	56 (43.4)	110 (43.5)	
Unknown	7	5	10	
RISS stage (%)				0.001
1	75 (27.9)	28 (25.0)	39 (24.8)	
2	140 (52.0)	52 (46.4)	80 (51.0)	
3	54 (20.1)	32 (28.6)	38 (24.2)	
Unknown	68	22	106	

Abbreviations: 17p‐, deletions of chromosome 17p; 1q21+, 1q21 gain/amplification; CD38, CD38 monoclonal antibody; ECOG, Eastern Cooperative Oncology Group; IMiDs, immunomodulatory agents; ISS stage, International Staging System; PI, proteasome inhibitors; RISS stage, Revised International Staging System; t(4;14), translocation between chromosome 4 and chromosome 14; t(14;16), translocation between chromosome 14 and chromosome 16.

### 3.1 Efficacy and Survival Outcome for Induction Treatment

#### 3.1.1 Response to Induction

A markedly higher percentage of patients attaining stringent complete remission (sCR)/complete remission (CR) was observed in the IMiDs plus PI cohort and CD38 monoclonal antibody‐based induction cohort compared to patients in the IMiDs or PI‐based induction cohort or conventional therapy cohort (*p* < 0.05). Moreover, a significantly greater number of patients achieved MRD negativity in the IMiDs plus PI cohort and CD38 monoclonal antibody‐based induction cohort compared to patients in the IMiDs or PI‐based induction cohort or conventional therapy cohort (*p* = 0.003) (Table 2). Given the imbalances observed among the four induction groups in Eastern Cooperative Oncology Group scores (ECOG), transplantation rates, Revised International Staging System (RISS) stages, we conducted multivariable analysis to control for the effects of these imbalanced baseline variables. The analysis confirmed that the choice of induction treatment independently impacted the likelihood of achieving an overall response (OR: ≥ VGPR) (Table 3).

**TABLE 2:** Response in different induction treatment group.

Best overall response to induction (%)	IMiDs + PI	CD38	IMiDs or PI	*p*
sCR + CR	149 (48.9)	69 (53.5)	65 (30.5)	< 0.001
VGPR	72 (23.6)	36 (27.9)	47 (22.1)	
PR	67 (22)	22 (17.1)	75 (35.2)	
MR	4 (1.3)	1 (0.8)	6 (2.8)	
SD	5 (1.6)	0 (0.0)	17 (8.0)	
PD	8 (2.6)	1 (0.8)	3 (1.4)	
≥ VGPR	221 (72.5)	105 (81.4)	112 (52.6)	< 0.001
MRD residual status				0.003
Negative	91 (61.1)	42 (77.8)	48 (49.5)	

Abbreviations: CD38, CD38 monoclonal antibody; CR, complete remission; IMiDs, immunomodulatory agents; MR, minimal response; PD, progressive disease; PI, proteasome inhibitors; PR, partial remission; sCR, stringent complete remission; SD, stable disease.

**TABLE 3:** Multivariable analysis.

	Adjusted OR	95% CI	*p*
Induction regimens			
IMiDs + PI	1		
CD38	2.34	(1.10–3.88)	0.023
IMiDs or PI	0.35	(0.22–0.55)	< 0.001
ECOG score			
0–1	1		
≥ 2	0.78	(0.51–1.20)	0.26
Transplantation			
Yes	1		
No	0.19	(0.43–1.18)	0.187
RISS stage			
I	1		
II	0.84	(0.52–1.37)	0.488
III	0.56	(0.31–1.02)	0.057

Abbreviations: CD38, CD38 monoclonal antibody; ECOG, Eastern Cooperative Oncology Group; IMiDs, immunomodulatory agents; PI, proteasome inhibitors; RISS stage, Revised International Staging System.

#### 3.1.2 Survival Outcome

##### 3.1.2.1 Overall Survival

**FIGURE 1:**
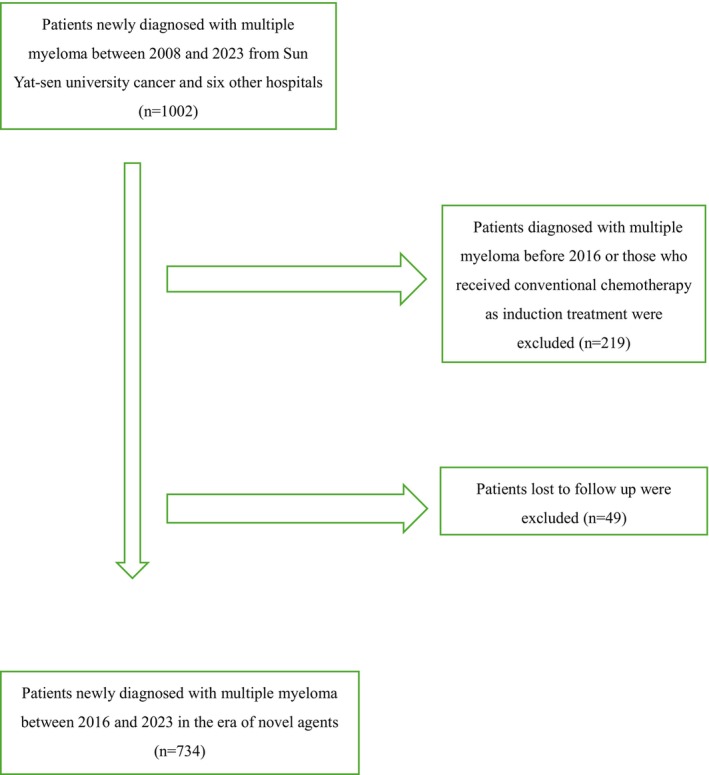
Flow chart of patients selection in our study.

The median OS was 70 months (95% CI: 58‐NR) for patients who received PI plus IMiDs, not reached for patients who received CD38 monoclonal antibody‐based regimens, and 67 months (95% CI: 52‐NR) for patients who received IMiDs or PI. Patients in the IMiDs plus PI group (*p* = 0.0424) and the CD38 group (*p* = 0.0051) exhibited a significantly better OS compared to those in the IMiDs or PI group. No significant difference in OS was observed between patients in the CD38 group and those in the IMiDs plus PI group (Figure 1A).

##### 3.1.2.2 Progression‐Free Survival

The median PFS was 54 months (95% CI: 43‐NR) for patients in the IMiDs plus PI group, 44 months (95% CI: 44‐NR) for patients in the CD38 group, and 44 months (95% CI: 38–54) for patients in the IMiDs or PI group. Similar to the trend observed in OS, a significantly superior PFS was observed in patients who received the CD38 monoclonal antibody (*p* = 0.0012) or IMiDs plus PI (*p* = 0.0377) compared to those who received either IMiDs or PI alone. Additionally, there was a trend toward improved PFS in the CD38 group compared to the IMiDs plus PI group (*p* = 0.0507) (Figure 1B).

### 3.2 The Impact of the Number of HRA on Prognosis

In this study, we divided patients into three groups based on the number of HRA. There were 327 patients without HRA, 156 patients with 1 HRA, and 46 patients with two or more HRAs. According to the baseline characteristics for patients in these three groups, those with HRAshad a more advanced International Staging System (ISS) stage, a more advanced Revised International Staging System (RISS) stage, a higher proportion of bone marrow plasma cells (BMPC), elevated levels of lactate dehydrogenase (LDH), and lower levels of hemoglobin (HGB) compared to those in the standard risk group (Table 4). This indicates that HRA is associated with a higher tumor burden, which may explain the worse prognosis for patients with HRA in the MM population.

**TABLE 4:** Baseline characteristics of patients without HRA, those with 1 HR ≥ 2 HRA.

	Without HRA	With 1 HRA	With ≥ 2 HRA	*p*
*n*	327	156	46	
Drug (%)				0.717
PI plus IMiDs	166 (50.8)	72 (46.2)	25 (54.3)	
CD38‐based	65 (19.9)	38 (24.4)	10 (21.7)	
PI or IMiDs	96 (29.4)	46 (29.5)	11 (23.9)	
Sex (%)				0.351
Female	140 (42.8)	60 (38.5)	23 (50.0)	
Male	187 (57.2)	96 (61.5)	23 (50.0)	
Age				0.77
≥ 65	105 (32.1)	55 (35.3)	16 (34.8)	
< 65	222 (67.9)	101 (64.7)	30 (65.2)	
MM type				0.189
IgA	54 (17.4)	36 (25.4)	12 (26.1)	
IgG	135 (43.5)	61 (43.0)	22 (47.8)	
IgD	6 (1.9)	6 (4.2)	3 (6.5)	
Light chain only	74 (23.9)	24 (16.9)	5 (10.9)	
Non‐secretory	39 (12.6)	14 (9.9)	4 (8.7)	
Biclonal	2 (0.6)	1 (0.7)	0 (0.0)	
Unknown	17	14	0	
ECOG score (%)				0.616
0–1	203 (64.4)	101 (68.2)	27 (61.4)	
≥ 2	112 (35.6)	47 (31.8)	17 (38.6)	
Unknown	12	8	2	
Transplantation (%)				0.931
Yes	72 (22.0)	32 (20.5)	10 (21.7)	
No	255 (78.0)	124 (79.5)	36 (78.3)	
ISS stage (%)				< 0.001
1	129 (40.3)	37 (24.2)	7 (15.2)	
2	79 (24.7)	43 (28.1)	10 (21.7)	
3	112 (35.0)	73 (47.7)	29 (63.0)	
Unknown	7	3	0	
RISS stage (%)				< 0.001
1	117 (37.7)	17 (11.5)	0 (0.0)	
2	165 (53.2)	83 (56.1)	14 (34.1)	
3	28 (9.0)	48 (32.4)	27 (65.9)	
Unknown	17	8	5	
BMPC%				0.036
< 50	241 (90.6)	109 (82.0)	35 (83.3)	
≥ 50	25 (9.4)	24 (18.0)	7 (16.7)	
Unknown	61	23	4	
FLCr (%)				0.381
< 100	198 (73.3)	80 (67.2)	29 (76.3)	
≥ 100	72 (26.7)	39 (32.8)	9 (23.7)	
Unknown	57	37	8	
LDH (%)				0.014
≥ 250	33 (10.1)	25 (16.0)	11 (23.9)	
< 250	294 (89.9)	131 (84.0)	35 (76.1)	
Cr (%)				0.698
≥ 177	42 (12.8)	21 (13.5)	8 (17.4)	
< 177	285 (87.2)	135 (86.5)	38 (82.6)	
Ca (%)				0.073
≥ 2.75	24 (7.3)	13 (8.3)	8 (17.4)	
< 2.75	303 (92.7)	143 (91.7)	38 (82.6)	
HGB (%)				0.004
≥ 100	176 (53.8)	65 (41.7)	15 (32.6)	
< 100	151 (46.2)	91 (58.3)	31 (67.4)	

Abbreviations: 17p‐, deletions of chromosome 17p; 1q21+, 1q21 gain/amplification; BMPC%, bone marrow plasma cell percentage; CD38, CD38 monoclonal antibody; ECOG, Eastern Cooperative Oncology Group; FLCr, involved free light chain/uninvolved free light chain ratio; HGB, hemoglobin; IMiDs, immunomodulatory agents; ISS stage, International Staging System; LDH, lactate dehydrogenase; PI, proteasome inhibitors; RISS stage, Revised International Staging System; t(4;14), translocation between chromosome 4 and chromosome 14; t(14;16), translocation between chromosome 14 and chromosome 16.

**FIGURE 2:**
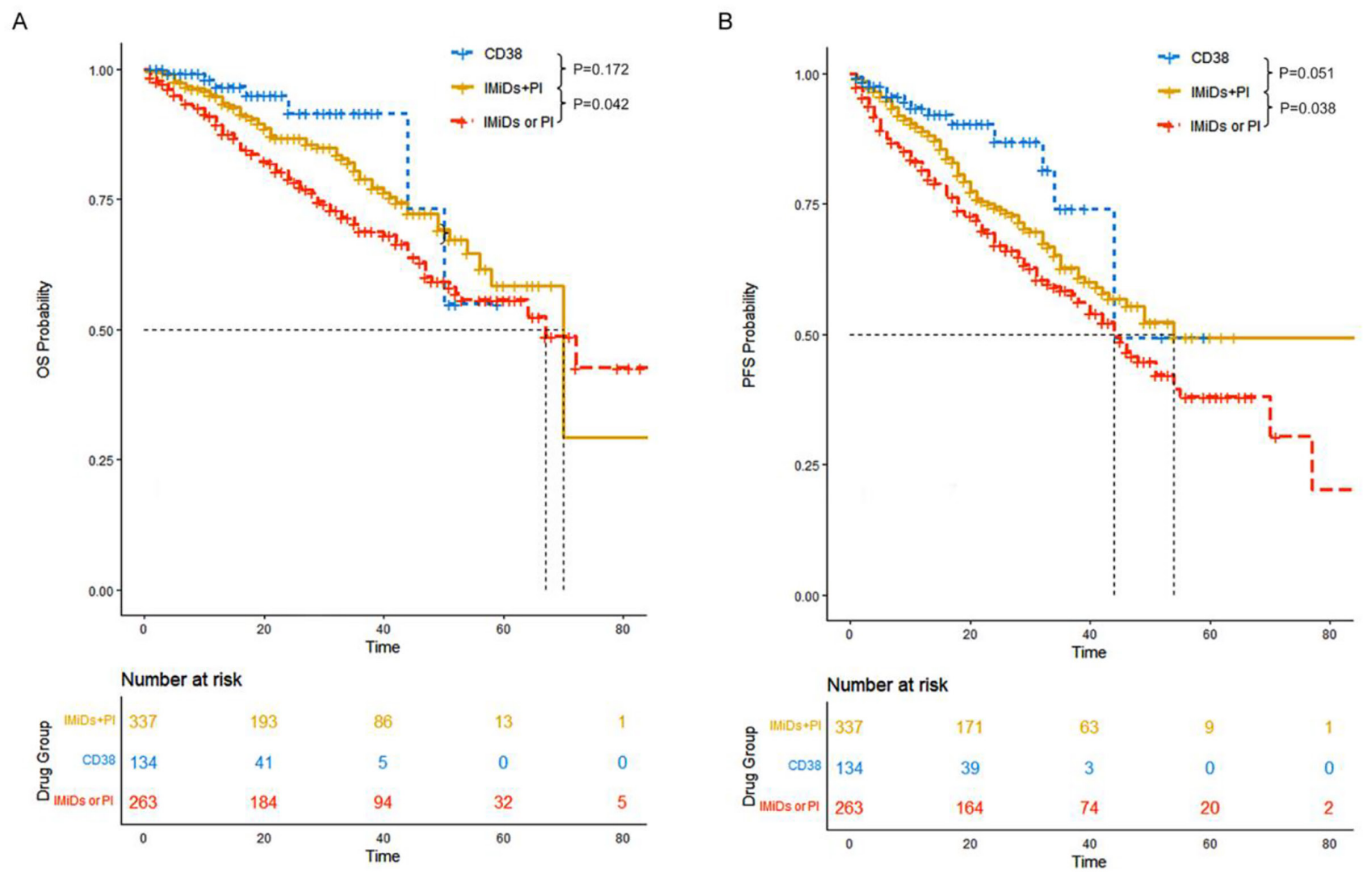
Overall survival (OS) and Progression free survival (PFS) of all patients. (A) OS. (B) PFS.

The median OS was 87 months (95% CI: NR‐NR) for patients without HRA, 64 months (95% CI: 42‐NR) for patients with 1 HRA, and 32 months (95% CI: 20‐NR) for patients with ≥ 2 HRA. Patients in the standard risk group had a significantly superior OS compared to those with 1 HRA (*p* = 0.0004) and those with ≥ 2 HRAs (*p* < 0.0001). Additionally, having 2 HRAs conferred a worse prognosis compared to having 1 HRA (*p* = 0.0368) (Figure 2A).

The median PFS was 55 months (95% CI: 46‐NR) for standard risk patients, 39 months (95% CI: 29–45) for patients with 1 HRA, and 20 months (95% CI: 17‐NR) for patients with ≥ 2 HRAs. Both 1 HRA (*p* = 0.0003) and 2 HRAs (*p* < 0.0001) were associated with a significantly inferior PFS outcome compared to 0 HRA. Moreover, there was a trend toward inferior PFS for patients with 2 HRAs compared to those with 1 HRA (*p* = 0.0787) (Figure 2B).

### 3.3 Whether CD38 Monoclonal Antibody, Transplantation, and MRD Negativity Could Overcome the Poor Prognosis Conferred by HRA

**FIGURE 3:**
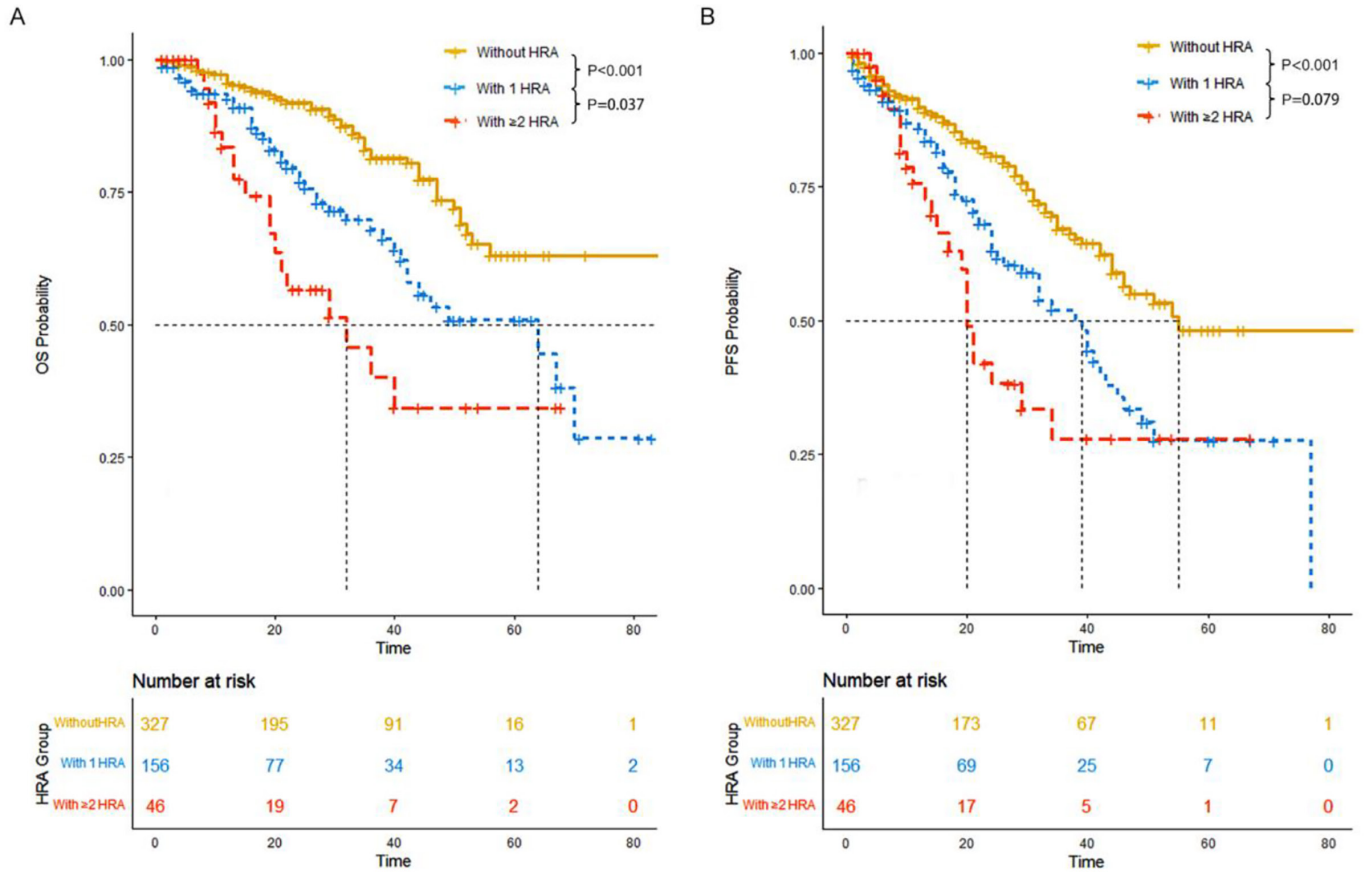
Overall survival (OS) and progression‐free survival (PFS) of patients without high‐risk cytogenetic abnormalities (HRAs), those with 1 HRA, and those with ≥ 2 HRA. (A) OS of patients without HRAs, those with 1 HRA, and those with ≥ 2 HRA. (B) PFS of patients without HRAs, those with 1 HRA, and those with ≥ 2 HRA.

Considering the reduction in OS and PFS for patients carrying HRA, we explored the efficacy of current treatment paradigms for these patients. Among patients without HRA, those in the CD38 group or the PI plus IMiDs group exhibited superior OS and PFS outcomes compared to those in the IMiDs or PI group. There was confirmed evidence for improved OS in patients who received the CD38 monoclonal antibody or the IMiDs plus PI combination. However, in the 1 HRA group, there was no significant OS and PFS advantage for the CD38 group or the IMiDs plus PI group, suggesting the limited efficacy of current novel agents in overcoming the poor prognosis associated with HRA. Similarly, patients with 2 HRA had shorter OS and PFS, and neither the CD38 monoclonal antibody nor the IMiDs plus PI combination significantly improved survival outcomes (Figure 3; Table 5).

**TABLE 5:** Summary of the median OS and PFS of patients.

	Patients group	Median OS	Median PFS
Induction	CD38	70 months [95% CI: 58‐not reached (NR)]	54 months (95% CI: 43‐NR)
	IMiDs + PI	NR	44 months (95% CI: 44‐NR)
	IMiDs or PI	67 months (95% CI: 52‐NR)	44 months (95% CI: 38–54)
HRA	Without HRA	87 months (95% CI: NR‐NR)	55 months (95% CI: 46‐NR)
	1 HRA	64 months (95% CI: 42‐NR)	39 months (95% CI: 29–45)
	2 HRA	32 months (95% CI: 20‐NR)	20 months (95% CI: 17‐NR)
1q21+	1q21+ only	64 months (95% CI: 42‐NR)	38 months (95% CI: 32–49)
	1q21+ plus 17p‐	22 months (95% CI: 15‐NR)	19 months (95% CI: 14‐NR)
	1q21+ plus t(4;14)	29 months (95% CI: 10‐NR)	29 months (95% CI: 9‐NR)

Abbreviations: 1q21+, 1q21 gain/amplification; CD38, CD38 monoclonal antibody; HRAs, high‐risk cytogenetic abnormalities; IMiDs, immunomodulatory agents; OS, overall survival; PFS, progression‐free survival; PI, proteasome inhibitors.

**FIGURE 4:**
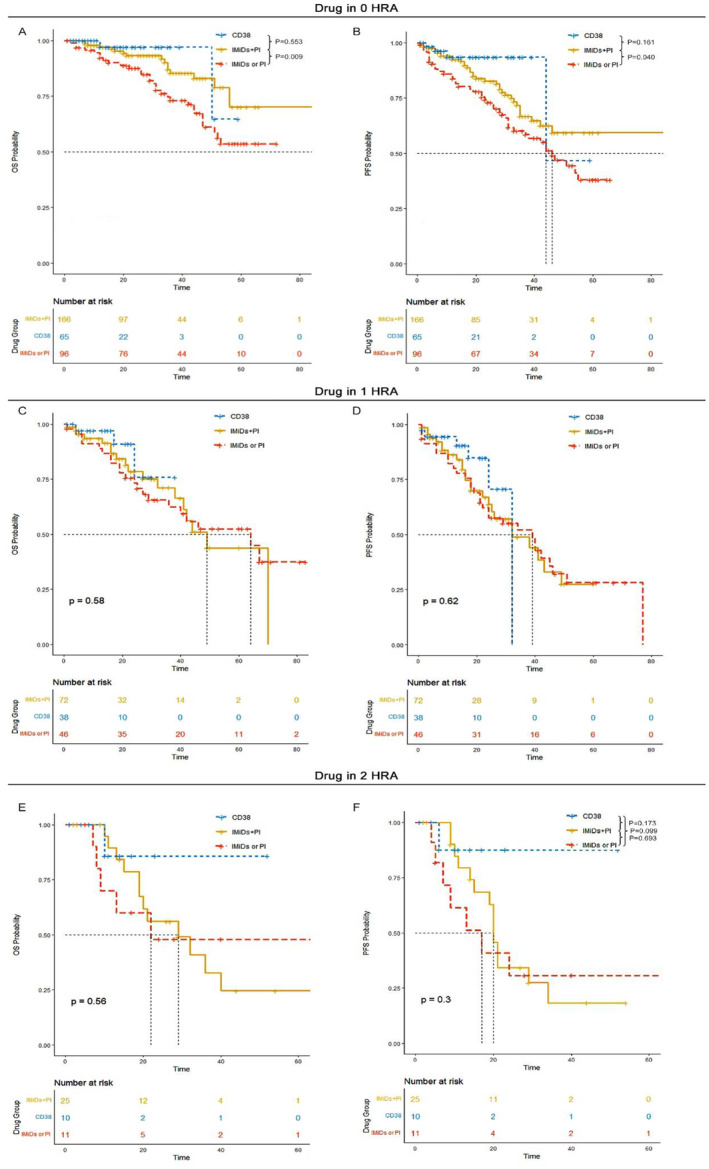
Overall survival (OS) and progression‐free survival (PFS) of different induction treatment for patients without high‐risk cytogenetic abnormalities (HRAs), those with 1 HRA, and those with ≥ 2 HRA. (A,B) OS and PFS of different induction treatment in 0 HRA group. (C,D) OS and PFS of different induction treatment in 1 HRA group. (E,F) OS and PFS of different induction treatment in ≥ 2 HRA group.

We further investigated the efficacy of transplantation in improving survival outcomes for patients carrying 1 HRA or ≥ 2 HRA. The results showed that although transplantation was effective in improving survival outcomes for standard risk patients, its efficacy was still limited in improving OS and PFS for patients carrying HRA (Figure 4A–F).

Moreover, we tested whether patients who achieved MRD negativity could overcome the poor prognosis associated with HRA. The results indicated that the value of MRD negativity was still limited for patients carrying HRA (Figure 4G–L).

### 3.4 1q21 Gain/Amplification Only versus Concomitant 1q21+ and Other HRA

1q21 gain/amplification (1q21+) has been recognized as a distinct entity among HRAs in MM. In recent years, numerous studies have investigated the significance of 1q21 in this disease [10, 11]. 1q21+ has been consistently associated with a poor prognosis in MM. Additionally, the co‐occurrence of 1q21+ with 17p deletion (17p‐) or t(4;14) is frequently observed in patients with MM. However, it remains unclear whether concurrent presence of 1q21 confers a worse prognosis than 1q21+ alone. Therefore, in our study, we compared the OS and PFS outcomes of 129 patients with 1q21 only, 14 patients with 1q21+ plus 17p‐, and 19 patients with 1q21+ plus t(4;14).

**FIGURE 5:**
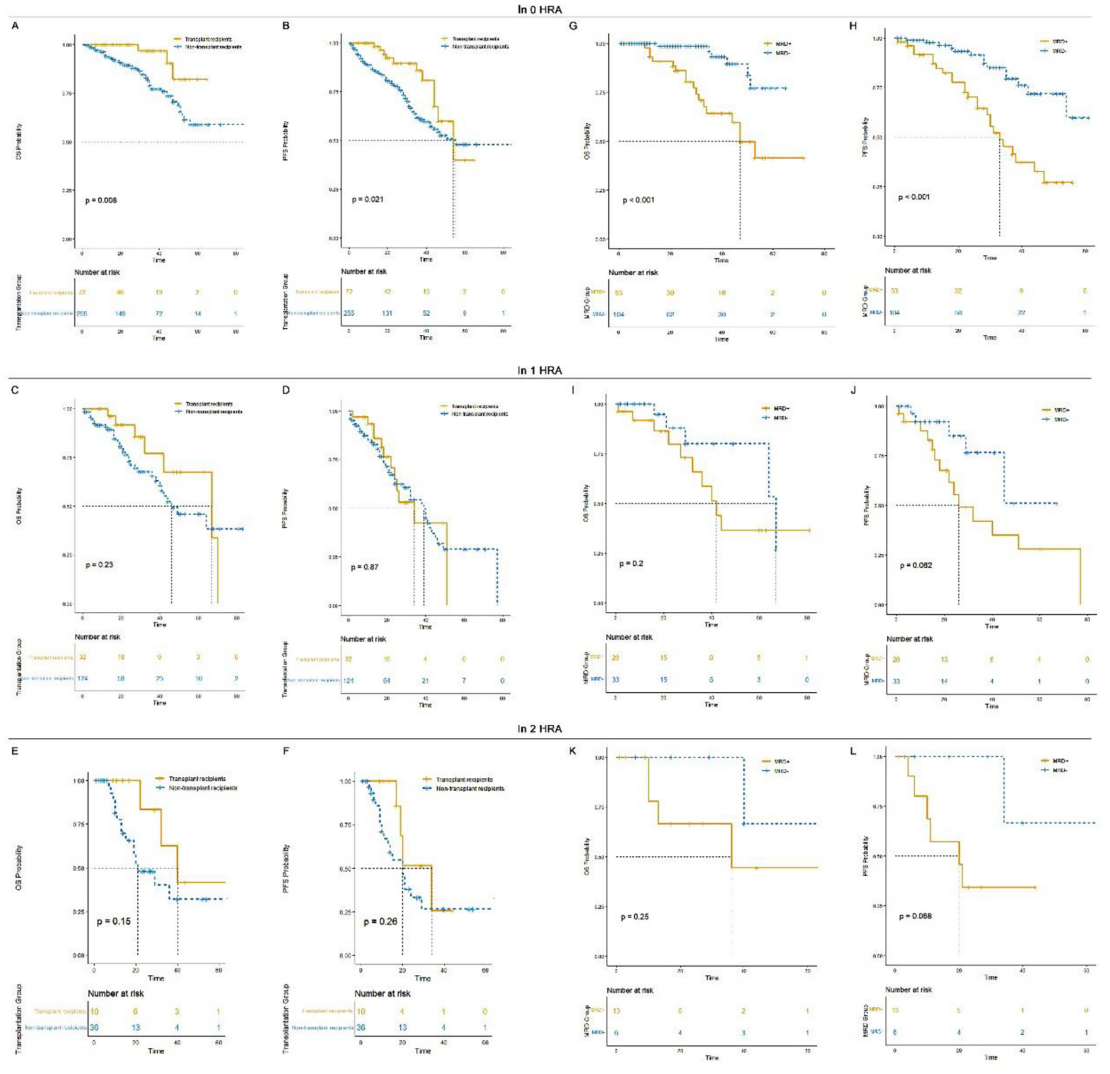
Overall survival (OS) and progression‐free survival (PFS) of transplant recipients versus non‐transplant recipients and MRD+ versus MRD− for patients without high‐risk cytogenetic abnormalities (HRA), those with 1 HRA, and those with ≥ 2 HRA. (A, B) Transplant recipients versus non‐transplant recipients for patients without HRA. (C, D) Transplant recipients versus non‐transplant recipients for patients with 1 HRA. (E, F) Transplant recipients versus non‐transplant recipients for patients with ≥ 2 HRA. (G, H) MRD+ versus MRD− for patients without HRA. (I, J) MRD+ versus MRD− for patients with 1 HRA. (K, L) MRD+ versus MRD− for patients with ≥ 2 HRA.

The median OS was 64 months (95% CI: 42‐NR) for patients with 1q21 only, 22 months (95% CI: 15‐NR) for those with 1q21+ plus 17p‐, and 29 months (95% CI: 10‐NR) for patients with 1q21+ plus t(4;14). Similarly, the median PFS was 38 months (95% CI: 32–49) for patients with 1q21+ only, 19 months (95% CI: 14‐NR) for those with 1q21+ plus 17p‐, and 29 months (95% CI: 9‐NR) for patients with 1q21+ plus t(4;14). There was a significantly better OS and PFS for patients with 1q21+ only compared to those with 1q21+ in combination with other HRAs (Figure 5A,B).

## 4 Discussion

We conducted this retrospective study to investigate the efficacy of current induction treatments and the impact of HRAs in a real‐world setting. The data for our study were collected from seven hospitals across China, spanning from north to south, providing a comprehensive overview of the current treatment paradigm for NDMM in China.

In our study, the use of CD38 monoclonal antibody and the combination of IMiDs plus PI provided a superior OS and PFS advantage, as well as a deeper response, compared to the use of IMiDs or PI alone. This finding is consistent with previous studies [12]. Additionally, the inclusion of CD38 monoclonal antibody in frontline therapy demonstrated a superior PFS advantage over IMiDs plus PI, although there was no significant difference in OS between the two treatments. These results were also confirmed in the large‐scale Phase III GRIFFIN trial [2]. This suggests a need for more effective drugs that could further prolong OS for NDMM in the future.

According to the mSMART guidelines, double‐hit MM is considered an ultra‐high‐risk category. In our study, we found that patients with ≥ 2 HRAs had worse survival outcomes compared to those with 1 HRA and those without HRA, suggesting the need for tailored treatment strategies for each group.

Within each HRA group, we assessed the impact of current frontline treatments on survival outcomes. While CD38 monoclonal antibody and the combination of IMiDs plus PI significantly improved OS and PFS for NDMM patients without HRA, their efficacy was limited in patients with 1 HRA and ≥ 2 HRA. In other retrospective studies, patients with a higher number of HRA also exhibited a worse prognosis [2, 13, 14].

Additionally, we examined whether transplantation and achieving MRD negativity could mitigate the poor prognosis associated with HRA. Unfortunately, our results indicated that neither transplantation nor the achievement of MRD negativity could completely overcome the adverse prognosis conferred by HRA. They only partly mitigated the poor survival outcomes in MM patients carrying HRA, which was confirmed in another retrospective study [7].

**FIGURE 6:**
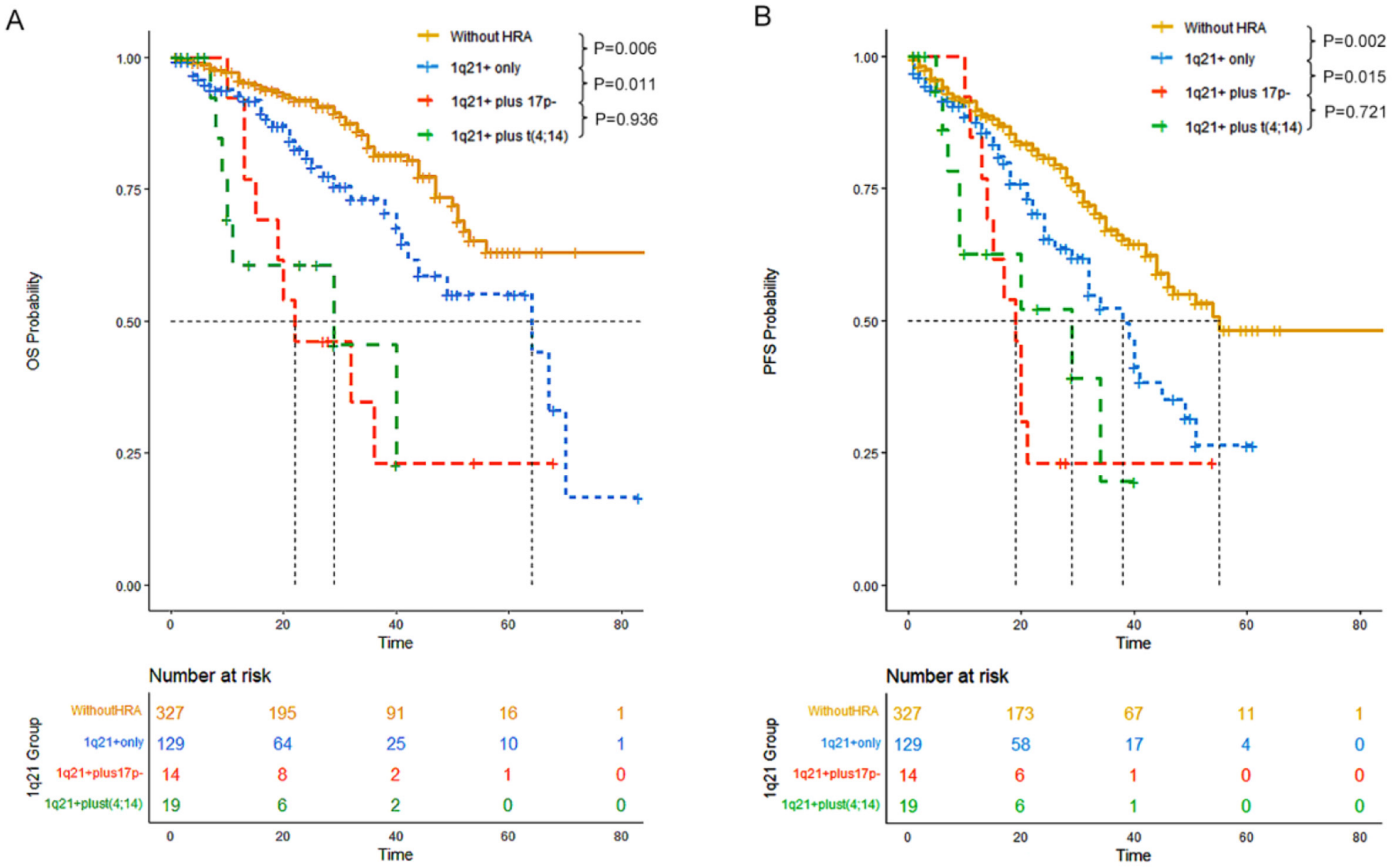
Overall survival (OS) and progression‐free survival (PFS) of patients without high‐risk cytogenetic abnormalities (HRAs), those with 1q21 gain/amplification (1q21+) only, those with 1q21+ plus del17p and those with 1q21+ plus t(4;14). (A) OS. (B) PFS.

For patients carrying 1q21 gain/amplification, those with 1q21+ in conjunction with other HRA had significantly inferior OS and PFS outcomes compared to those with 1q21+ alone. Future studies should thoroughly investigate the implications of concomitant 1q21+ and other HRA to better understand their impact on prognosis and to develop more effective treatment strategies (Figure 6).

Our study had several limitations. First, it was a retrospective study. Second, the number of patients in the subgroup analysis was relatively small. Third, the toxic events associated with the induction regimens were not recorded in detail.

Our study confirmed the advantage of using two or more novel agents in the treatment of MM. Additionally, the challenge posed by HRA was also highlighted in this retrospective study. In the future, we aim to compare more advanced therapies, such as CAR‐T cells and bispecific antibodies (BsAb), for NDMM. We hope that these advanced therapies will help overcome the poor prognosis associated with HRA.

## Author Contributions

**Dong Liang:** writing – original draft (lead). **Xiaojin Li:** writing – review and editing (equal). **Shenrui Bai:** data curation (supporting), resources (supporting). **Qiaoli Wang:** visualization (equal). **Min Zeng:** resources (equal). **Demei Feng:** data curation (supporting), resources (supporting). **Bo Lu:** data curation (supporting), resources (supporting). **Xiaoqing Li:** data curation (equal), resources (equal). **Zhiqiang Sun:** data curation (supporting), resources (supporting). **Jianyun Li:** data curation (equal), resources (equal). **Huanhuan Zhou:** data curation (equal), resources (supporting). **Jialu Zhang:** data curation (supporting), resources (supporting). **Xiaoqin Chen:** data curation (supporting), resources (supporting). **Zhongjun Xia:** data curation (supporting), resources (supporting). **Yang Liang:** conceptualization (supporting), resources (supporting). **Hua Wang:** conceptualization (equal), resources (equal).

## Ethics Statement

This study was approved by Institutional Ethical Review Board of Sun Yat‐Sen university cancer center. The International Conference on Harmonization's Good Clinical Practice standards and the 1964 Helsinki Declaration and its later revisions served as the foundation for the study's methodology.

## Conflicts of Interest

The authors declare no conflicts of interest.

## Data Availability

Data can be obtained from the corresponding author upon reasonable request.
