# Correction to “Clinical Outcome of Induction Treatment in the Era of Novel Agents and the Impact of the Number of High‐Risk Cytogenetic Abnormalities (HRA) on Prognosis of Patients With Newly Diagnosed Multiple Myeloma (NDMM): Insights From a Multicenter Study”

**DOI:** 10.1002/cam4.70406

**Published:** 2024-11-10

This corrects the article “Clinical Outcome of Induction Treatment in the Era of Novel Agents and the Impact of the Number of High‐Risk Cytogenetic Abnormalities (HRA) on Prognosis of Patients With Newly Diagnosed Multiple Myeloma (NDMM): Insights From a Multicenter Study” PMID: 39422477 PMCID: PMC11487677 DOI: 10.1002/cam4.70270

The correct affiliations for Xiaojin Li are as follows:

2. Faculty of Environmental Science and Engineering, Kunming University of Science and Technology, Kunming, Yunnan, China

3. The Affiliated Hospital of Yunnan University, Kunming, Yunnan, China

The correct title of Table 4 is “Baseline characteristics of patients without HRA, those with 1 HRA and those with ≥ 2 HRA”

We apologize for these errors.

